# Clinical experience regarding the diagnostic value of segment-by-segment coronary computed tomography angiography in comparison with that of invasive coronary angiography

**DOI:** 10.1590/0100-3984.2021.0092

**Published:** 2022

**Authors:** Rafael Mansur Souto, Alair Augusto Sarmet Moreira Damas dos Santos, Marcelo Souto Nacif

**Affiliations:** 1 Hospital Universitário Antônio Pedro - Universidade Federal Fluminense (HUAP-UFF), Niterói, RJ, Brazil.

**Keywords:** Multidetector computed tomography, Coronary angiography/methods, Coronary artery disease/diagnostic imaging, Tomografia computadorizada multidetectores, Angiografia coronária/métodos, Doença da artéria coronária/diagnóstico por imagem

## Abstract

**Objective:**

To compare the degree of coronary stenosis (≥ 50% luminal narrowing) determined by coronary computed tomography angiography (CCTA) with that determined by invasive coronary angiography (ICA), using segment-by-segment analysis.

**Materials and Methods:**

This was a retrospective study of the records of patients who underwent CCTA and ICA between January 2014 and June 2018 at a general hospital in Brazil. Receiver operating characteristic curve analysis was applied, and the areas under the curve were used in order to assess the overall accuracy of the methods.

**Results:**

The degree of coronary stenosis was evaluated in a total of 844 arterial segments. The diagnostic performance of CCTA was good, with a sensitivity of 82.3%, a specificity of 96.4%, and a negative predictive value of 97.7% (95% CI: 96.5-98.5). In the segment-by-segment analysis, CCTA had excellent accuracy for the left main coronary artery and for other segments.

**Conclusion:**

In clinical practice at general hospitals, CCTA appears to have diagnostic performance comparable to that of ICA.

## Abstract (pt)

**Objetivo:**

O objetivo do estudo é comparar os graus de estenose coronariana (≥ 50% de redução luminal) determinados pela tomografia computadorizada e pelo cateterismo, utilizando uma análise segmento a segmento.

**Materiais e Métodos:**

Estudo retrospectivo conduzido em pacientes que foram submetidos a tomografia computadorizada e a cateterismo, de janeiro de 2014 a junho de 2018, em um hospital geral. A análise da curva característica de operação do receptor foi utilizada para a análise da acurácia.

**Resultados:**

Na avaliação dos vasos, em um total de 844 segmentos, o desempenho da tomografia computadorizada foi bom, com sensibilidade de 82,3%, especificidade de 96,4% e valor preditivo negativo de 97,7% (IC 95%: 96,5-98,5). Na análise segmento a segmento, o tronco da coronária esquerda, assim como outros segmentos, apresentaram excelente acurácia.

**Conclusão:**

A tomografia computadorizada mostrou bom desempenho diagnóstico quando comparada com o cateterismo na prática diária de um hospital geral.

## INTRODUCTION

Cardiovascular diseases are responsible for the death of more than 17.9 million people annually, accounting for 31% of all deaths worldwide^(1)^. Eighty percent of those deaths are caused by acute myocardial infarction or stroke^(1,2)^. The diagnosis of coronary artery disease is important for the initiation of specific therapy and the prevention of ischemic events^(3,4)^.

Approximately two decades ago, noninvasive evaluation of the coronary arteries using coronary computed tomography angiography (CCTA) became possible. The effectiveness of this method has been demonstrated in various studies, in which its correlation with invasive coronary angiography (ICA) has been examined. The use of CCTA is very important to exclude or detect coronary artery disease, even at subclinical levels^(5-7)^. This method has a negative predictive value (NPV) of 96-100%, making it reliable for the exclusion of coronary artery stenosis^(6)^.

To our knowledge, there have been no studies involving segment-by-segment and per-patient analyses of the correlation between CCTA and ICA findings in a hospital setting in Brazil. The objective of this study was to evaluate the degree of coronary stenosis (≥ 50% luminal narrowing) determined by segment-by-segment analysis at a general hospital, comparing CCTA and ICA. We sought to determine whether CCTA and ICA are similar in terms of their ability to predict coronary artery disease in daily clinical practice at a general hospital in Brazil.

## MATERIALS AND METHODS

This was a retrospective, cross-sectional, observational study of data related to patients who underwent CCTA and ICA involving cardiac catheterization, between January 2014 and June 2018, at the Complexo Hospitalar de Niterói, in the city of Niterói, Brazil. The Complexo Hospitalar de Niterói is a tertiary care hospital, operated by Universidade Federal Fluminense, with a 24-h emergency department that is a referral center for trauma cases. The Research Ethics Committee of Universidade Federal Fluminense approved the study (Reference no. 85407818.4.0000.5243). Because of the retrospective nature of the study, the requirement for written informed consent was waived.

**Figure 1:**
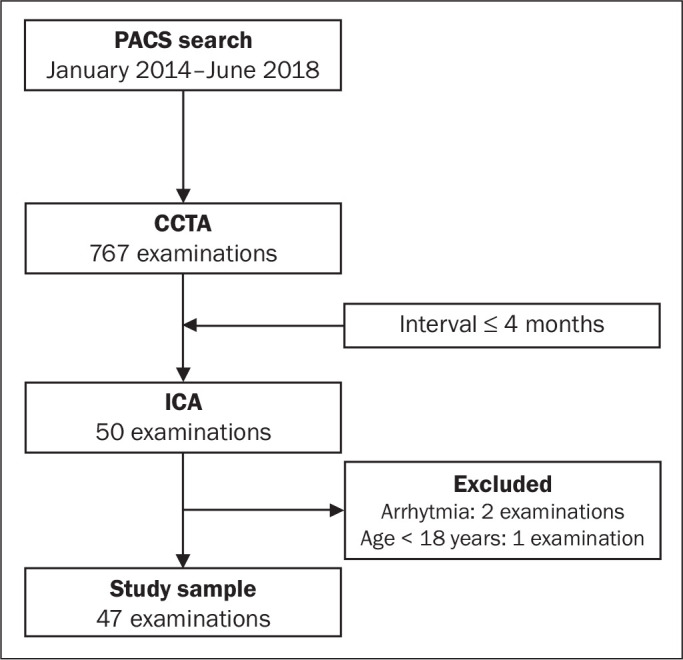
Flow chart of the selection of imaging examinations for inclusion in the study.

Imaging studies in the Digital Imaging and Communications in Medicine format were identified by a search of the Picture Archiving and Communication System of the hospital. We reviewed the records of all adult patients (≥ 18 years of age) who, at the request of their physicians, had undergone ICA < 4 months after CCTA, to monitor chronic coronary artery disease. We included only imaging studies that were of diagnostic quality, with no artifacts that would render analysis unviable (e.g., pronounced arrhythmia, involuntary movements, and respiratory motion). A flow chart of the study selection process is displayed in Figure 1.

### CCTA protocol

All CCTA examinations were performed in a 64-slice CT scanner (Somatom Sensation 64; Siemens, Forchheim, Germany), using a specific electrocardiogram-gating protocol, before and after intravenous injection of contrast medium. If the heart rate was above 65 bpm and there were no contraindications to the use of metoprolol tartrate (e.g., asthma or difficult-to-control heart failure), it was prescribed at a dose of 5-30 mg. Prior to injection of the contrast medium, all of the patients were given sublingual isosorbide dinitrate (5 mg) for coronary vasodilation, except for the patients with contraindications to its use. Patients at risk for adverse reactions to contrast medium were submitted to a desensitization protocol: oral prednisone (20 mg every 6 h), starting 12 h before the procedure; and oral diphenhydramine (50 mg), at 1 h before the procedure.

Non-ionic contrast (60 mL, Henetix 350; Guerbet, Villepinte, France) was injected into an antecubital vein at 5 mL/sec, after which 20 mL of an isotonic saline solution (0.9% NaCl) were administered with a dual-syringe injection pump (Stellant; Medrad, Indianola, PA, USA). The contrast bolus trigger was used in order to determine the timing of CCTA acquisition, allowing the arrival of the contrast medium in the middle ascending aorta to be noted.

### ICA protocol

All ICA examinations were performed through transradial access with a 6F sheath. The angiography system used (Artis zee; Siemens Healthineers, Erlangen, Germany) had a 17-in. intensifier. A minimum of eight X-ray projections were acquired for the study of the coronary arteries.

### CCTA imaging analysis

Two radiologists, with 5 and 17 years of experience in cardiac imaging, respectively, evaluated the images and accompanying reports. The radiologists interpreted the images by consensus, using axial source images, thin-slab maximum intensity projections, and multiplanar reconstruction on an image processing workstation (Leonardo; Siemens Healthineers). Coronary segments were identified by using the segmentation protocol devised by Raff et al.^(8)^. For each segment, significant stenosis was defined as luminal narrowing ≥ 50%.

### ICA imaging analysis

Images from ICA examinations were stored digitally in multiple views and subsequently analyzed by a cardiologist who was blinded to the CCTA results. Coronary segmentation followed the same protocol used in the CCTA analysis.

**Figure 2:**
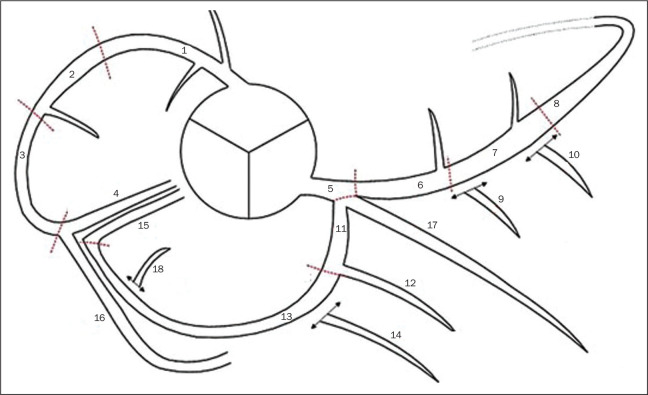
Coronary segmentation. 1, RCAp; 2, RCAm; 3, RCAd; 4, PDA; 5, LMCA; 6, LADp; 7, LADm; 8, LADd; 9, Dg1-LAD; 10, Dg2-LAD; 11, LCxp; 12, Mg1-LCx; 13, LCxd; 14, Mg2-LCx; 15, PDA-LCx; 16, PLV; 17, diagonal branch; 18, PLV-LCx.

On the basis of the segmentation protocol devised by Raff et al.^(8)^, we defined 21 coronary segments (Figure 2): left main coronary artery (LMCA); diagonal branch; left anterior descending artery (LAD); proximal, middle, and distal LAD (LADp, LADm, and LADd, respectively); first, second, and third diagonal LAD (Dg1-LAD, Dg2-LAD, and Dg3-LAD, respectively); left circumflex artery (LCx); proximal and distal LCx (LCxd and LCxp, respectively); three marginal LCx (Mg1-LCx, Mg2-LCx, and Mg3-LCx); right coronary artery (RCA), proximal, middle, and distal RCA (RCAp, RCAm, and RCAd, respectively); marginal RCA (Mg1-RCA); posterior descending artery (PDA); and posterior left ventricular artery (PLV). In relation to the Raff et al.^(8)^ protocol, we added the Mg1-RCA and the Dg3-LAD. The PDA and PLV originated from the RCA or, in a few cases, from the LCx (PDA-LCx and PLV-LCx).

### Statistical analysis

Continuous variables are expressed as means ± standard deviations, whereas categorical variables are expressed as absolute and relative frequencies. Considering ICA as the gold-standard method of imaging, we calculated the accuracy, sensitivity, specificity, positive predictive value (PPV), and NPV of CCTA, with 95% confidence intervals (95% CIs).

We assessed the performance of CCTA in the identification of ≥ 50% luminal narrowing, relative to that of ICA, by using receiver operating characteristic (ROC) curve analysis. Findings from both examinations were analyzed segment by segment and per patient. Segments with ≥ 50% and < 50% luminal narrowing, as determined by anatomical evaluation via ICA, served as true-positive and true-negative markers, respectively.

The ROC analysis was applied to the categorical responses, and the overall accuracy of each analysis was assessed by calculating the area under the curve (AUC). We considered AUCs ≥ 0.5 to < 0.7 to be indicative of poor agreement between the performance of CCTA and that of ICA in evaluating stenosis grading, whereas we considered AUCs ≥ 0.7 to < 0.9 to be indicative of good agreement and AUCs ≥ 0.9 to 1.0 to be indicative of excellent agreement.

Multiple comparisons with kappa tests were performed to assess the level of agreement between the ICA and CCTA analyses in a segment-by-segment mode and in a per-patient mode. The MedCalc statistical software package, version 14.8.1.0 for Windows (MedCalc Software, Ostend, Belgium) was used for the statistical analyses. Values of *p* < 0.05 on two-tailed tests were considered to be significant.

## RESULTS

We reviewed data from 50 patients who underwent ICA and CCTA during the study period. Two patients were excluded due to excessive arrhythmia-related artifacts, and one patient was excluded for being under 18 years of age. Therefore, the final sample comprised 47 patients. The mean age was 69.1 ± 12.1 years (range, 18-95 years), and 36 (76.6%) of the patients were male.

### Per-patient analysis

Of the 47 patients evaluated, 11 (23.4%) did not present coronary stenosis ≥ 50% on CCTA or ICA (a true-negative result), one (2.1%) showed coronary stenosis ≥ 50% only on ICA (a false-negative result), two (4.2%) showed coronary stenosis only on CCTA (a false-positive result), and 33 (70.2%) showed coronary stenosis on both methods (a true-positive result). The overall prevalence of coronary stenosis was 72.3%.

For CCTA, the PPV was 94.3% and the NPV was 91.7%. In the ROC curve analysis, the AUC for CCTA, in comparison with ICA, was 90.8% (95% CI: 78.8-97.3). We found CCTA to have a sensitivity of 97.1% (95% CI: 84.7-99.99) and a specificity of 84.6% (95% CI: 54.5-98.0).

### Analysis of all segments

**Figure 3:**
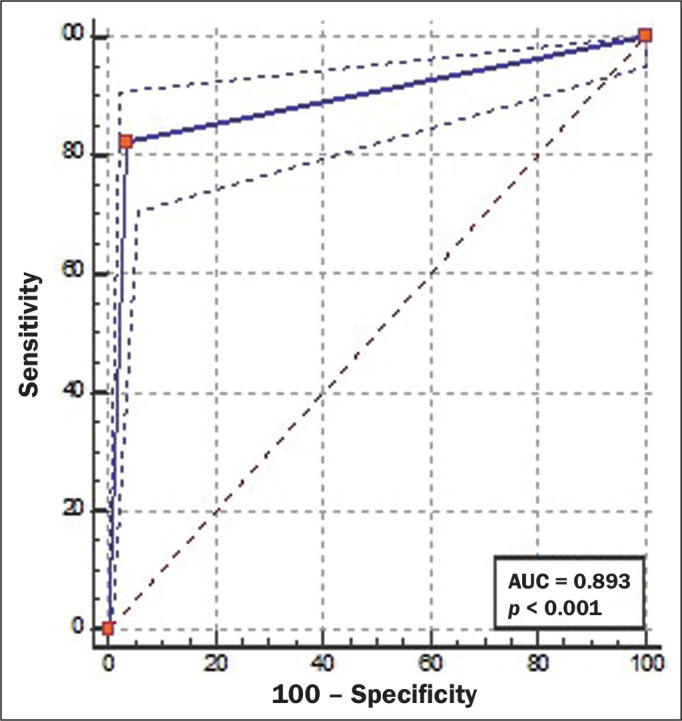
ROC curve analysis of CCTA and ICA for all 844 segments evaluated.

A total of 844 coronary segments were included in the analysis. As can be derived from Figure 3, CCTA showed an accuracy of 89.3% (95% CI: 87.1-91.3), with a sensitivity of 82.3% (95% CI: 73.2-89.3), a specificity of 96.4% (95% CI: 94.8-97.6), a PPV of 74.6% (95% CI: 66.7-81.2), and an NPV of 97.7% (95% CI: 96.5-98.5). For the identification of ≥ 50% luminal narrowing, we obtained true-negative results for 721 segments (85.4%), false-negative results for 27 (3.2%), false-positive results for 17 (2.0%), and true-positive results for 79 (9.0%), the difference being significant (*p* < 0.0001).

### Segment-by-segment analysis

Results of the segment-by-segment analysis are presented in Table 1. The diagonal branch was omitted from the analysis because it was not present in any of the patients in our sample. The Dg3-LAD, Mg1-RCA, and Mg3-LCx corresponded to 46 segments each, none of which showed ≥ 50% luminal narrowing on CCTA or ICA. The PDA-LCx and PLV-LCx corresponded to three segments each. None of the PLV-LCx showed ≥ 50% luminal narrowing on either examination, and one PDA-LCx segment showed ≥ 50% luminal narrowing only on CCTA. For all of these segments, it was not possible, with the software used for the statistical analyses, to calculate the ROC curve. Therefore, none of these 144 segments appear in Table 1.

**Table 1:** Concordance between CCTA and ICA findings for stenosis (≥ 50% luminal narrowing), by segment.

Segment	Normal		Stenosis		AUC (95% CI)	P
	ICA	CCTA	ICA	CCTA		
	(n = 604)	(n = 595)	(n = 96)	(n = 105)		
	n (%)	n (%)	n (%)	n (%)		
LMCA	45 (96)	45 (96)	2 (4.3)	2 (4.3)	1.00 (0.93-1.00)	< 0.0001
LADp	34 (72)	33 (70)	13 (28)	14 (30)	0.88 (0.75-0.96)	< 0.0001
LADm	26 (55)	22 (47)	21 (45)	25 (53)	0.92 (0.81-0.98)	< 0.0001
LADd	46 (98)	45 (96)	1 (2.1)	2 (4.3)	0.98 (0.90-1.00)	—
Dg1-LAD	35 (75)	41 (82)	12 (26)	6 (13)	1.00 (0.93-1.00)	< 0.0001
Dg2-LAD	44 (96)	43 (94)	2 (4.3)	3 (6.5)	0.99 (0.90-1.00)	< 0.0001
LCxp	37 (79)	36 (77)	10 (21)	11 (23)	0.80 (0.65-0.90)	0.0002
LCxd	39 (83)	38 (81)	8 (17)	9 (19)	0.84 (0.70-0.93)	0.0001
Mg1-LCx	44 (94)	44 (94)	3 (6.4)	3 (6.4)	1.00 (0.93-1.00)	< 0.0001
Mg2-LCx	46 (98)	46 (98)	1 (2.1)	1 (2.1)	1.00 (0.92-1.00)	—
RCAp	39 (83)	34 (72)	8 (17)	13 (28)	0.94 (0.82-0.99)	< 0.0001
RCAm	40 (85)	38 (81)	7 (15)	9 (19)	0.90 (0.77-0.96)	< 0.0001
RCAd	42 (92)	42 (89)	4 (8.5)	5 (11)	0.99 (0.90-1.00)	< 0.0001
PDA	41 (93)	42 (96)	3 (6.8)	2 (4.5)	0.83 (0.69-0.93)	0.0455
PLV	43 (98)	4 (100)	1 (2.3)	0 (0.0)	0.50 (0.35-0.65)	—

The accuracy of CCTA was best for the LMCA (AUC = 1.00; 95% CI: 0.93-1.00), the Mg1-LCx (AUC = 1.00; 95% CI: 0.93-1.00), the Dg1-LAD (AUC = 1.00; 95% CI: 0.93-1.00), the RCAd (AUC = 0.99; 95% CI: 0.90-1.00), and the Dg2-LAD (AUC = 0.99; 95% CI: 0.90-1.00). There was no statistically significant difference for the PLV, Mg2-LCx, or LADd.

## DISCUSSION

This study revealed good agreement between CCTA and ICA in the identification of ≥ 50% luminal narrowing at a general hospital in Brazil. We found that CCTA showed accuracy exceeding that obtained in randomized studies^(5-7)^.

The NPV of CCTA for all 844 segments examined in the present study was 97.7%, which is comparable to values obtained in other studies, such as that conducted by Mahdavi et al.^(9)^, who reported an NPV of 97.2% for 628 segments in 47 patients. In a CCTA validation study, Budoff et al.^(7)^ obtained an NPV of 99.0% for the identification of ≥ 50% luminal narrowing in 910 vessels. We found that the PPV of CCTA was lower than was its NPV, as was also found by Scheffel et al.^(10)^. In our sample, the NPV was less than 100% because some patients did not have class I recommendations for CCTA, reflecting the fact that our assessment was performed in an everyday clinical setting, without strict application of exclusion criteria, which may have led to an overestimation in the quantification of obstructive calcified plaques. Taken together, however, these findings demonstrate that CCTA performs well for the exclusion of coronary artery disease, thus minimizing the risk of unnecessary invasive procedures^(11-13)^.

The AUC of 0.91 obtained for CCTA in our per-patient analysis is similar to the 0.96 obtained by Budoff et al.^(7)^, who applied more exclusion criteria. As in the present study, Chow et al.^(14)^ showed that CCTA had excellent sensitivity and a high NPV in comparison with ICA.

There have been few studies involving segment-by-segment analysis of the accuracy of CCTA. We found that the accuracy of CCTA was better for proximal segments (i.e., the LMCA, LADp, and LADm) than for the smaller-caliber segments, which is consistent with previous reports that luminal evaluation can be hampered in the latter^(11,12,15)^. In the present study, CCTA revealed ≥ 50% stenosis in nearly all of the coronary segments so identified by ICA, the exceptions being the LMCA, Dg1-LAD, Mg1-LCx, RCAp, and PLV. It has been suggested that CCTA overestimates the degree of stenosis^(14)^, potentially explaining the greater proportion of lesions with ≥ 50% stenosis on CCTA, which did not, in all cases, match that obtained with ICA.

Our study has some limitations. First, we used ICA findings as the reference, rather than using the fractional flow reserve, magnetic resonance imaging findings, or echocardiographic data, any of which might have permitted a more definitive quantification of the lesions. In addition, reconstruction techniques and stenosis quantification methods used in CCTA analysis, such as multiplanar reconstruction, were not applied to the X-rays obtained during catheterization, which could have led to the overestimation or underestimation of some stenosis values. However, the fact that the study was carried out at a general hospital, outside of an academic environment, shows the reality of CCTA in daily practice, which is extremely important knowledge for cardiologists who work in hospitals in Brazil.

## CONCLUSION

In regular clinical practice at a general hospital in Brazil, CCTA shows diagnostic performance in the identification of stenosis comparable to that of ICA, as determined by segment-by-segment and per-patient analyses. That finding corroborates those obtained in CCTA validation studies.
